# Overexpression of the cancer-related DUSP1 gene in human obesity and its modulation by physical exercise

**DOI:** 10.1186/2051-1426-2-S1-P5

**Published:** 2014-02-24

**Authors:** Abdelkrim Khadir, Ali Tiss, Jehad Abubaker, Mohamed Abu-farha, Said Dermime, Mohammed Dehbi

**Affiliations:** 1 Deptartment of Biomedical Research, Dasman Diabetes Institute, Dasman, Kuwait

## 

Obesity is a major risk factor for metabolic diseases and cancer. Lifestyle interventions in obesity reduce the risk for cancer. Aberrant regulation of dual specificity protein phosphatase 1 (DUSP1) was linked to obesity and cancer representing an attractive therapeutic target and to study the relationship between obesity and cancer. Here, we investigated the effects of a 3-month physical exercise protocol on the expression of DUSP1 in obese humans and analyzed correlations between DUSP1 levels and study population characteristics. Compared to lean subjects, DUSP1 mRNA and protein are highly expressed in the adipose tissue of obese subjects with a concomitant decrease of phospho-p38 MAPK and PGC-1α; the two downstream targets of DUSP1 and an increase in the levels of IL-6 and TNF-α inflammatory markers. High levels of DUSP1 correlated positively with BMI, percent body fat, blood pressure, triglycerides and C-peptide (P<0.05). Our data provide for the first time evidence that physical exercise significantly attenuated DUSP1 expression with an increase in PGC-1α, and improvement of the inflammatory and stress responses. Given that both DUSP1 and PGC-1α have been linked to cancer, their modulation by physical exercise can be considered as a non-pharmacological approach and thereby, mitigating the risk for cancer development in obese population.

